# p62-Keap1-NRF2-ARE Pathway: A Contentious Player for Selective Targeting of Autophagy, Oxidative Stress and Mitochondrial Dysfunction in Prion Diseases

**DOI:** 10.3389/fnmol.2018.00310

**Published:** 2018-10-04

**Authors:** Syed Zahid Ali Shah, Deming Zhao, Tariq Hussain, Naveed Sabir, Mazhar Hussain Mangi, Lifeng Yang

**Affiliations:** National Animal Transmissible Spongiform Encephalopathy Laboratory, Key Laboratory of Animal Epidemiology and Zoonosis of Ministry of Agriculture, College of Veterinary Medicine and State Key Laboratory of Agrobiotechnology, China Agricultural University, Beijing, China

**Keywords:** prion protein scrapie (PrP^Sc^), extracellular plaques, hydrogen peroxide toxicity, mitochondrial dysfunction, nuclear factor erythroid 2-related factor 2 (NRF2)

## Abstract

Prion diseases are a group of fatal and debilitating neurodegenerative diseases affecting humans and animal species. The conversion of a non-pathogenic normal cellular protein (PrP^c^) into an abnormal infectious, protease-resistant, pathogenic form prion protein scrapie (PrP^Sc^), is considered the etiology of these diseases. PrP^Sc^ accumulates in the affected individual’s brain in the form of extracellular plaques. The molecular pathways leading to neuronal cell death in prion diseases are still unclear. The free radical damage, oxidative stress and mitochondrial dysfunction play a key role in the pathogenesis of the various neurodegenerative disorders including prion diseases. The brain is very sensitive to changes in the redox status. It has been demonstrated that PrP^c^ behaves as an antioxidant, while the neurotoxic prion peptide PrP^Sc^ increases hydrogen peroxide toxicity in the neuronal cultures leading to mitochondrial dysfunction and cell death. The nuclear factor erythroid 2-related factor 2 (NRF2) is an oxidative responsive pathway and a guardian of lifespan, which protect the cells from free radical stress-mediated cell death. The reduced glutathione, a major small molecule antioxidant present in all mammalian cells, and produced by several downstream target genes of NRF2, counterbalances the mitochondrial reactive oxygen species (ROS) production. In recent years, it has emerged that the ubiquitin-binding protein, p62-mediated induction of autophagy, is crucial for NRF2 activation and elimination of mitochondrial dysfunction and oxidative stress. The current review article, focuses on the role of NRF2 pathway in prion diseases to mitigate the disease progression.

## Introduction

Transmissible spongiform encephalopathies (TSEs), also termed prion diseases, are a group of rare and progressive neurodegenerative disorders affecting several mammalian species including humans. Animal prion diseases are bovine spongiform encephalopathies (BSE) in cattle, scrapie disease in sheep and goats, and chronic wasting disease in elk and wild deer. Human prion diseases include kuru, Creutzfeldt-Jakob disease (CJD), Gerstmann-Straussler-Scheinker syndrome (GSS) and Fatal Familial Insomnia (FFI; Nakagaki et al., 2013; Puig et al., 2016; Shah et al., 2017d). An important event in prion diseases is the continuous and constant conversion of normal cellular alpha-helical prion protein (PrP^c^) into an abnormal beta-sheet rich, protease-resistant, misfolded, and aggregated isoform termed prion protein scrapie (PrP^Sc^; Pan et al., 1993). The molecular mechanisms and signaling cascades resulting in neuronal inflammation and death are unclear, and therefore there is no effective treatment available for this fatal neurodegenerative disorder (Moreno et al., 2013; Shah et al., 2017b).

Conclusive evidence suggests that oxidative stress, which originated from the damage caused by free radicals and various lipids, proteins, carbohydrates, and DNA, is dynamically implicated in the cellular apoptosis during Alzheimer’s disease (AD; Smith et al., 1997, 1998; Pappolla et al., 1998) and several other neurodegenerative disorders, such as amyotrophic lateral sclerosis (ALS) and Huntington’s disease (HD; Beal et al., 1997; Browne et al., 1999; Cookson and Shaw, 1999). Furthermore, various studies have suggested that the normal alpha helical cellular prion protein (PrP^c^) may be actively involved in the cellular response and resistance mechanisms against oxidative stress (Brown et al., 1997a, 1998b; Choi et al., 1998; Wong et al., 1999). Brown and colleagues further confirmed that the neurons and other brain resident cells, such as astrocytes obtained from mice deficient in PrP^c^, were more sensitive to the damage produced by oxidative stress (Brown et al., 1997b, 1998a). This evidence suggests that the presence and quantity of PrP^c^ is crucial for cell survival, and the continuous conversion of the normal cellular prion to misfolded PrP^Sc^ would lead to a pronounced oxidative stress in prion-afflicted individuals.

Mitochondria have an essential role in many neurodegenerative disorders including prion diseases (Li et al., 2017; Shah et al., 2017c). Mitochondria are the vital regulators of apoptosis, a key aspect of neurodegeneration (Toyama et al., 2016). Mitochondrial DNA mutations and the production of reactive oxygen species (ROS) both contribute towards cell death and degeneration. Molecular research on prion disease cell model suggest that the mitochondrial dysfunction and endoplasmic reticulum stress leading to enhanced ROS production are interconnected events resulting from the accumulation of misfolded prion proteins (Ferreiro et al., 2006, 2008a,b). Reduced expression of the respiratory chain enzymes, such as a flavoprotein NADH dehydrogenase (also known as ubiquinone) 1 beta subcomplex subunit 8 (NDUFB8; also termed as complex I subunit), succinate dehydrogenase B (SDHB; also called complex II subunit), ubiquinol-cytochrome c reductase core protein 2 (UQCRC2; also known as complex III subunit), cyclooxygenase 2 (COX2; also called complex IV subunit), and adenosine triphosphate 50 (ATP50; also known as complex V subunit), has been observed in the brains of prion-affected individuals. Moreover, marked neuronal apoptosis led to suppressed expression of voltage-dependent anion channel (VDAC) and ATP5H, which further enhanced the oxidative stress damage (Frau-Méndez et al., 2016). Guentchev et al. (2000) demonstrated that peroxinitrite generation marker, nitrotyrosine (NT) and an enzyme leading to the formation of antioxidant molecules, heme-oxygenase-1 (HO-1), were upregulated in the mice model of prion diseases. Thus, therapies that target the fundamental mitochondrial processes, including energy metabolism or the production of free radicals, or the specific interactions between disease-causing proteins (PrP^Sc^) with mitochondria, hold enormous promise towards therapeutic intervention in prion diseases.

Autophagy, also called autophagocytosis, is an essential and important protein degrading pathway and a vital segment of the innate cellular immune system, which is activated in the affected brain tissue of prion diseases (Khan et al., 2017; Lai et al., 2018). The major endogenous source of oxidative stress is the association of mitochondria-resident electron transport chain in the pathogenesis of neurodegenerative diseases including prion diseases. Impaired autophagy augments mitochondrial dysfunction, and its association with the pathogenic misfolded PrP^Sc^ significantly contributes towards increasing the oxidative stress generation and decreasing the production of ATP (Khan et al., 2017; Li et al., 2017). The cytosol-resident ubiquitin-binding protein p62 is a protein known to intercede the degradation of aggregated PrP^Sc^ in the brain (Khan et al., 2017). The levels of p62 protein are elevated during prion diseases (Homma et al., 2014). The p62-Kelch-like ECH associated protein 1-nuclear factor erythroid 2-related factor 2 (p62-Keap1-NRF2) axis is linked to the induction of partial and selective autophagy through its interaction with ubiquitin and LC3 component of autophagy. This signaling cascade is regulated by post-translational modifications such as chronological phosphorylation and ubiquitination of the p62 (Katsuragi et al., 2016). Combating the oxidative stress and maintaining cellular redox homeostasis is important for cell survival. A fundamental pathway in maintaining the cellular redox homeostasis is the NRF2- antioxidant response element (ARE) signaling cascade. The NRF2-ARE signaling cascade induces the expression of cytoprotective and antioxidative proteins and enzymes that play a central role in mitigating cellular stress caused by the production of pro-inflammatory cytokines and oxidative stress stimuli (Johnson et al., 2008). Enhanced expression of NRF2 has been confirmed in both *in vivo* and *in vitro* studies, and it has been demonstrated that the NRF2 diminishes various neuron damaging changes ranging from the lipid peroxidation (Ansari et al., 2011) and excitotoxic signaling cascades (Li et al., 2007), to calcium metabolism pathways (Lee et al., 2003) and various mitochondrial alterations (Ludtmann et al., 2014). In all the brain-resident cells, the astrocytes have an elevated level of NRF2 protein (Williamson et al., 2012), while on the other hand, they have a low PrP^c^ expression. Additionally, it has been shown that inhibiting the activity of NRF2 increases the expression of PrP^c^ in an astrocyte-dominant cell line (Brown, 2004). Regardless of this contrary repressing role played by NRF2 on the PrP^c^ expression, Cichon and Brown (2014) showed that the expression of PrP^c^ in response to oxidative stress was very significant and the increased PrP^c^ levels were greater in the NRF2 overexpressing cells. This indicates that the signaling pathways that enhance the cellular PrP^c^ expression in response to oxidative stress damage are independent of NRF2 activation, but they are dependent on the oxidative stress. Recently, it has been demonstrated that the activation of autophagic flux and the NRF2-ARE pathway protected against acrylonitrile-induced neurotoxicity in primary rat astrocytes (Yang et al., 2018). In the current review article, we will focus on the modulation of the p62-Keap1- NRF2-ARE pathway for selective targeting of autophagy, oxidative stress, and mitochondrial dysfunction in prion diseases as therapeutic intervention strategy.

## The Structure and Regulation of NRF2

**Figure 1:**
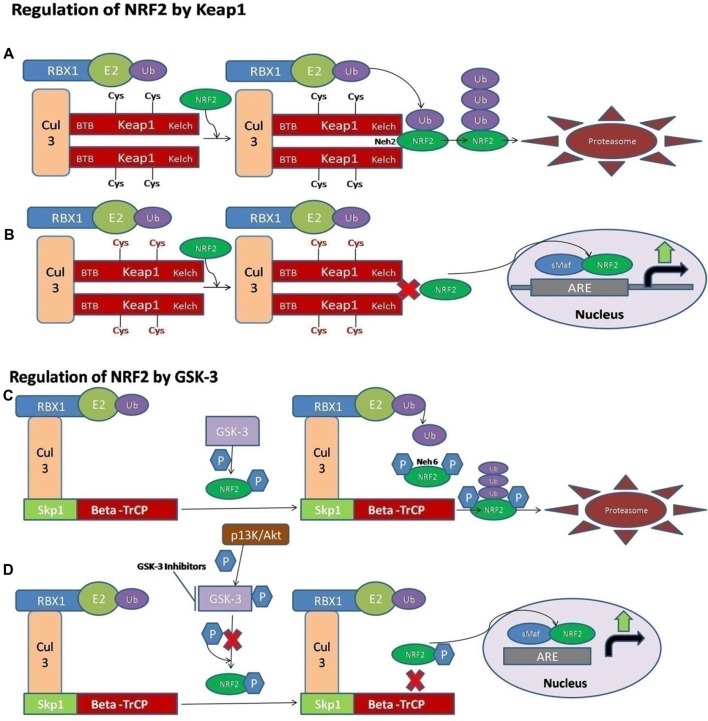
A schematic diagram of nuclear factor erythroid 2-related factor 2 (NRF2) activation via Kelch-like ECH associated protein 1 (Keap1) and GSK-3. **(A)** The ligase complex Cullin 3 (Cul3) RING-box 1 (RBX1) E3 binds to the adaptor protein Keap1 via Tramtrack and Bric-a-Brac (BTB) domain. The Keap1 protein is ultimately able to interact with the Neh2 domain of NRF2 and that allows the ubiquitination and degradation of the NRF2 in the cytoplasm. **(B)** The electrophilic compounds modify the specific sensors known as the Cys sensor residues located on Keap1, leading to various conformational alterations that prevent NRF2 ubiquitination. Finally, NRF2 is accumulated and translocated to the nucleus, and a dimer is formed with the small musculoaponeurotic fibrosarcoma proteins (sMaf) to bind the antioxidant response element protein (ARE) regions in the DNA and mediate the upregulation of its target genes. **(C)** Alternatively, the NRF2 is activated via Glycogen synthase kinase-3 (GSK-3). The adaptor protein known as beta-transducin repeat containing protein (β-TrCP) binds to the complex of SCF ubiquitin ligase. Then, the GSK-3 protein phosphorylates NRF2 at the Neh6 domain. The phosphorylated form of NRF2 is predictable by the β-TrCP complex, which targets it for ultimate degradation. **(D)** Various signaling pathways, such as the PI3K/Akt pathway, are able to phosphorylate the GSK-3 protein and disrupt its activity, hence allowing the accumulation of NRF2.

NRF2 activity is tightly controlled and regulated in the mammalian cells. Under normal physiological conditions, the NRF2 is kept at low levels, as continuous degradation of NRF2 occurs in the ubiquitin proteasome system. There are a variety of ubiquitin ligase systems that are accountable for targeting and degradation of the NRF2 in the proteasome system. One of these ligase complex systems is the Cullin 3 (Cul3) RING-box 1 (RBX1) E3 ubiquitin ligase system. The Keap1 acts as a substrate for the ubiquitination of the NRF2 protein (Cullinan et al., 2004; Kobayashi et al., 2004; Zhang et al., 2004). The Keap1 protein is encoded by the Keap1 gene and it is located in the cytoplasm. Keap1 represses the activity of NRF2. Keap1 possesses distinct protein domains. The most important of them is the N-terminal Broad complex, Tramtrack and Bric-a-Brac (BTB) domain, which contains the residue Cys151—an important stress sensing cysteine. The second important domain is the intervening region (IVR) domain, containing two vital cysteine residues, Cys273 and Cys288. These are the second group of cysteines essential for stress sensing. A β-propeller structure is formed by a double glycine repeat (DGR) and C-terminal region (CTR) domain that is where Keap1 interacts with the NRF2 protein (Itoh et al., 1999; Holland and Fishbein, 2010). The Keap1 dimerization and binding to Cul3 occurs through the BTB domain (Furukawa and Xiong, 2005), whereas the Kelch domain of Keap1 is able to interact with the Neh2 domain of the NRF2 protein (Itoh et al., 1999; McMahon et al., 2006). A cyclic mechanism of ubiquitination and degradation takes place, where the NRF2 is first degraded, while the Keap1 is regenerated to keep the NRF2 in the suppressed status (Baird et al., 2013, 2014; Figure 1A). Keap1 is a very attractive drug target for oxidative stress conditions, and the absence of Keap1 activity will lead to non-sequestered and non-degraded NRF2 in the cytoplasm. The build-up of NRF2 in the cytoplasm will lead to its nuclear translocation and trigger the transcription of its target genes (Dinkova-Kostova et al., 2002; Eggler et al., 2005; Fourquet et al., 2010; McMahon et al., 2010; Saito et al., 2015). It is reported that under oxidative stress conditions, some Keap1 cysteine residues are chemically modified, and they prevent the ubiquitination and degradation of the NRF2 protein. This will allow the stabilization of NRF2 and the transcriptional triggering of the target genes affected by the NRF2 signaling (Holland and Fishbein, 2010; Figure 1B).

It has also been documented that some of the electrophilic lipids, for example, the subset of prostaglandins known as cyclopentenone prostaglandin, 15-deoxy-Δ12,14–prostaglandin J2 (that act as a study model to view the impact of lipid peroxidation products resulting from the reactions of ROS with the unsaturated fatty acids), are also able to trigger the NRF2 pathway by modifying the thiol groups of the Keap1 domain (Levonen et al., 2004). Another critical pathway or signaling mechanism by which the modification of the various cysteines in Keap1 leads to the suppressed degradation of NRF2 is through the decreased affinity of Keap1 for the Cul3 protein (Gao et al., 2007; Eggler et al., 2009).

Autophagy lowers the risk of neurodegenerative diseases including prion diseases. The ubiquitin-binding protein p62 is an important protein complex associated with the selective autophagy. The degradation of Keap1 depending on p62 is another method of the NRF2 pathway activation and cell protection (Taguchi et al., 2012). Park et al. (2015) showed that this mechanism occurs in response to cytotoxic stress caused by lipids. Additionally, this activation occurs by the sestrins as shown by Bae et al. (2013). Furthermore, p62 interacts with the NRF2-binding sites located on the Keap1 protein, thus simultaneously competing with the NRF2 protein, and ultimately preventing the degradation of the NRF2 protein (Jain et al., 2010; Komatsu et al., 2010; Lau et al., 2010). This condition is seen where the p62 protein is found in the phosphorylated form (Ichimura et al., 2013).

Recently, the important role of a new E3-ubiquitin ligase adaptor protein called the beta-transducin repeat containing protein (β-TrCP), was studied in combination with an Skp, Cullin, F-box containing complex known as the SCF ubiquitin ligase complex. All these reactions are Keap1-independent and are tightly regulated by the serine threonine protein kinase known as the glycogen synthase kinase-3 (GSK-3). The effect of GSK-3 phosphorylation is seen on the Neh6 domain of NRF2 and it ultimately targets the NRF2 for degradation by the proteasome system through the SCF/β-TrCP complex, and therefore it can restrain the NRF2 activity via this complex (Rada et al., 2011; Chowdhry et al., 2013; Figure 1C). The Keap1 protein acts as a main sensor for the electrophiles and oxidants, and the GSK-3/β-TrCP complex plays an important role in receptor-dependent signal transduction and modulates the levels of NRF2 protein in response to short-lived metabolic demands reactions (Cuadrado, 2015). This machinery could engage those signaling cascades that can take part in the GSK-3 regulation, such as the PI3K/Akt or WNT signaling cascades (Figure 1D).

## Oxidative Stress in Prion Diseases

**Figure 2:**
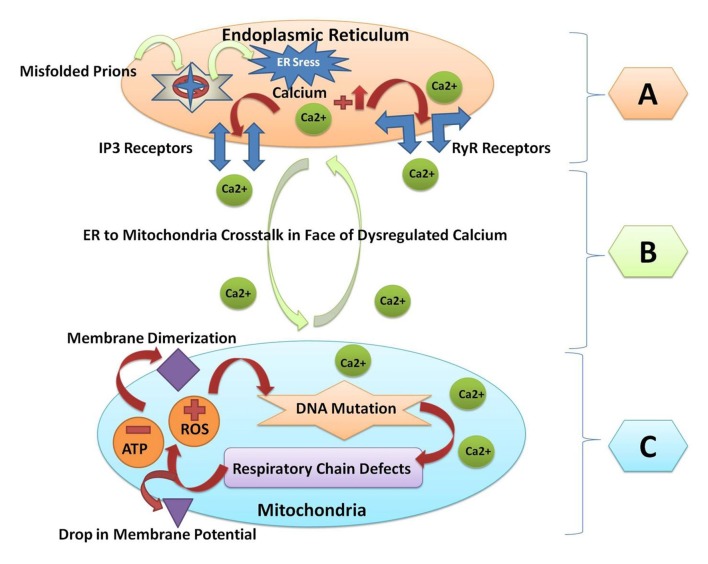
A Schematic diagram of crosstalk between endoplasmic reticulum and mitochondria resulting from prion fibrils accumulation within the brain. **(A)** Prion fibrils are accumulated in the endoplasmic reticulum (a major calcium storing organelle), and it activates ER stress. Dysregulated calcium will result in increased calcium flux in the lumen of the endoplasmic reticulum. Opening of the inositol receptors (IP3R) and ryanodine receptors (RyR) are located on the membrane of the endoplasmic reticulum as a result of dysregulated calcium levels. **(B)** Escape of calcium from the endoplasmic reticulum will result in the endoplasmic reticulum to mitochondria crosstalk, and unfolded protein response within the endoplasmic reticulum is triggered to restore the normal functions. **(C)** When the stress goes beyond the tolerable level, the reactive oxygen species (ROS) are produced within the mitochondria and the adenosine triphosphate (ATP) producing capacity is hampered. ROS will result in mitochondrial DNA mutation. Respiratory chain complex abnormalities will result in further production of ROS and decrease the ATP levels. Finally, the membrane dimerization and drop in membrane potential will result in the initiation of apoptosis and cell death.

All the major protein misfolding neurodegenerative diseases including prion disorders are characterized by the aggregation and accumulation of the misfolded/unfolded proteins within the brain of the affected individuals. In prion diseases, the central event is posttranslational modification of the normal PrP^c^ to an aberrant and conformationally distorted isoform known as prion protein scrapie (PrP^Sc^; Pan et al., 1993). A multifunctional organelle endoplasmic reticulum is a major cellular compartment accountable for the proper protein folding and processing. The accumulation of the misfolded prions leads to endoplasmic reticulum stress and dysregulated calcium signaling (Shah et al., 2015a,b). Calcium imbalance within the cell triggers the endoplasmic reticulum to mitochondrial crosstalk to counteract the stress produced by the misfolded proteins (Shah et al., 2017a). Mitochondrial dysfunction and production of ROS within the cell are general pathomechanisms that underlie prion diseases (Ferreiro et al., 2008a,b). All of these events can activate the oxidative stress reactions and impair the mitochondrial dynamics by increasing the peroxynitrite levels, and ultimately cause cellular injury via the formation of the hydroxyl radicals or via the nitration of the tyrosine residues on the proteins (Figure 2).

The oxidative stress can be defined as the loss of equilibrium between the ROS levels produced within the cell and the endogenous cellular potential to neutralize these harmful reactive intermediates, ultimately leading to unfavorable conditions that contribute to cellular death and damage (Betteridge, 2000). Superoxide dismutase (SOD2) plays an important role in scavenging the superoxides produced during the normal energy metabolism. SOD2-ablated mice treated with the external synthetic SOD2–catalase mimetics extended the lifespan by three folds and rescued the mice from spongiform encephalopathies (Melov et al., 2001). The overstimulation of NADPH oxidase activity has been observed in PrP106–126-infected neuroepithelial cells (Pietri et al., 2006). It has been demonstrated that the ROS production is divided into acute, adaptive, and chronic phases during the course of prion infection. In the initial stages of prion infection, the cell adapts to an increased amount of intracellular ROS production. The adaptation occurs very quickly within the overall cell population. This adaptive response is perhaps necessary for the maintenance of cell viability. Afterwards, the cells enter further into a progressive chronic phase of infection, in which the ROS response is very tightly kept below the level of damage to the cells. Finally, the protective adaptive responses fail in a large subpopulation of those cells that are chronically infected and will undergo apoptosis (Haigh et al., 2011). The elevated nitric oxide (NO) levels in the CNS are predominant features of prion diseases. The NO is produced by the enzymatic action of nitric oxide synthase (NOS). Park et al. (2011) demonstrated that the endothelial NOS levels were upregulated in the brains of the scrapie-infected mice. Our group previously showed that ferric ions and prion peptide PrP106–126 transform the alpha helical-rich cellular prion to an abnormal beta sheet-rich protease resistant isoform PrP^Sc^. The NADH concentrations were reduced by up to 60% in comparison to the normal non-infected cells. In other words, we can say that nearly 40% of the normal respiratory chain complexes were lost after treatment of prion-infected N2a cells with ferric ions (Yuan et al., 2013). Recently, Tahir et al. (2018) demonstrated that the reactive sulfur species in combination with the ROS and the reactive nitrogen species, which lead to multiple toxic insults, were observed in the brain of sCJD patients. The build-up of oxidative stress beyond the tolerable level within the cell will deregulate many cellular functions, such as the deregulation of energy-dependent metabolic pathways (Dringen et al., 2000), the impairment of energy-producing mitochondrial network (Gandhi and Abramov, 2012), the impairment of signal transduction (Apel and Hirt, 2004), the activation of the cell death pathways (Salganik, 2001), and the misfolding/aggregation of the pathogenic proteins (Bruijn et al., 1998; Valentine and Hart, 2003). The cerebral cortex of the sCJD patients and the prion-infected Syrian golden hamsters’ brain revealed increased oxidation, lipoxidation, glycoxidation and nitrative contents (Freixes et al., 2006; Pamplona et al., 2008). Therefore, the oxidative stress is implicated as one of the primary contributors to the quick onset and progression of sCJD disease pathogenesis.

## Mitochondrial Dysfunction in Prion Diseases

Mitochondrial dysfunction triggered by the abrupt aggregation and accumulation of misfolded/unfolded proteins is a major feature in all the neurodegenerative disorders (Figure 2; Shah et al., 2015b; Ansoleaga et al., 2016; Khan et al., 2017). Mitochondria are the main affected cellular organelle in prion diseases. It has been shown that the prion peptide PrP106–126 rapidly depolarizes the mitochondrial membranes in the human neuroblastoma cell line SH-SY5Y. The rapid depolarization of mitochondria results in the caspase activation and dysregulated calcium that ultimately raise the cellular calpain levels and trigger apoptosis. The combinatory caspase and calpain inhibitor treatment rescued cells from the prion peptide-induced apoptosis (O’Donovan et al., 2001). Hachiya et al. (2005) demonstrated that an impaired proteasome system leads to aberrant translocation of PrP^c^ to mitochondria and it triggers the apoptotic signaling. Similarly, Martin et al. (2007) showed that the levels of the coenzymes Q_9_ (CoQ_9_) and Q_10_ increased with the disease progression in BSE infected mice. The CoQ_9_- and CoQ_10_-mediated protective system combats the ROS, but this system seems to be insufficient to prevent the disease (Figure 2; Martin et al., 2007). The continuous fusion and fission reactions within the energy storing organelle mitochondria act as an adaptive response to both the internal and external cellular insults (Wai and Langer, 2016).

Mitochondrial impairments and production of ROS are vital factors in the pathogenesis of a number of neurodegenerative diseases including prion diseases. The increased activity of phospholipase D1 (PLD1) has been observed in scrapie-infected brains. Moreover, the concentration of mitochondrial-resident phospholipids, for instance a phospholipid containing choline, phosphatidylcholine and phospholipid containing ethanolamine, phosphatidylethanolamine, were both increased, and the content of phosphatidic acid, a product produced by PLD activity, was amplified in the specific mitochondrial membrane-obtained fractions (Jin et al., 2005). The mitochondrial fission reactions require the activation of the energy-dependent AMPK signaling (Toyama et al., 2016). Molecular research on class III histone deacetylase sirtuin 1 (Sirt1) established the link between prion diseases and autophagic mechanisms. We know that a suppressed or decreased mitophagy contributes to the build-up of defective and functionally impaired mitochondria in the cell. This will lead to an energy imbalance inside the cell. A number of research groups showed altered mitochondrial dynamics in various models of prion diseases (Choi et al., 2014; Ansoleaga et al., 2016; Frau-Méndez et al., 2016; Faris et al., 2017). Sirt1 plays an essential role in the initiation of autophagy and protection of neurons against mitochondrial damage produced by the cytotoxic prion proteins. Previously it had been demonstrated that this cytoprotection was principally linked to a decrease in the mitochondrial membrane potential value and also linked with the decrease in the PrP fragment (106–126)-induced apoptotic translocation of cell death related protein Bax into the mitochondria and subsequent release of cytochrome c from the mitochondria into the cytosol (Jeong et al., 2013). Similarly, a compound known to activate the Sirt1 protein activity, known as resveratrol, played a crucial role in the rescue of cellular injury by prevention of PrP fragment (106–126)-induced neuronal apoptotic signaling. The neurotoxic events were blocked by the induction of autophagic mechanism via the autophagy-lysosome signaling pathway (Jeong et al., 2012). Siskova et al. (2011) demonstrated that the mitochondrial impairments are believed to occur due to the inhibition or modification of the respiratory chain complexes (Complex-IV and Complex-II) rather than the removal of the mitochondrial DNA content. Our group demonstrated that c-Abl tyrosine kinase plays a crucial role during prion infection. The c-Abl-BIM signaling cascades resulted in cell death via mitochondrial dysfunction in rat hippocampal neurons and N2a cells. Interestingly, the silencing of c-Abl tyrosine kinase resulted in the alleviation of oxidative stress-mediated mitochondrial dysfunction and cell death (Pan et al., 2014).

## Activation of NRF2 in Prion Diseases

**Figure 3:**
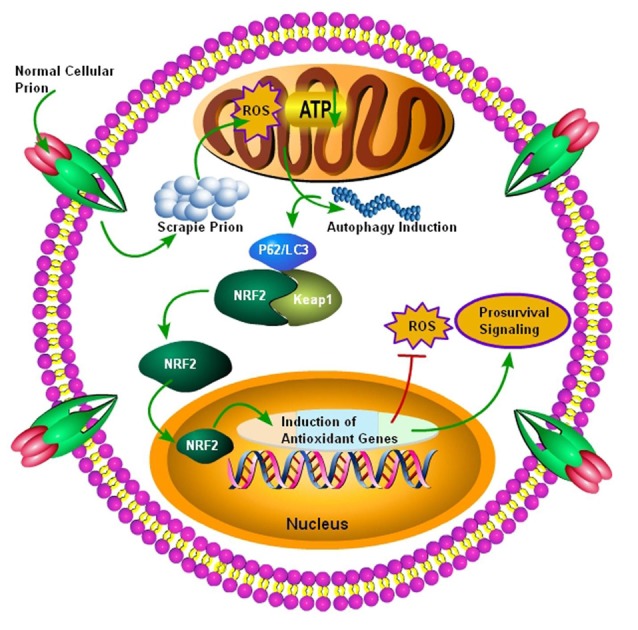
Autophagy mediated activation of NRF2 Pathway. Initially normal cellular prion protein (PrP^c^) is converted into scrapie prion protein (PrP^Sc^) which is ultimately transported for degradation. The failure of ubiquitin-proteasome system results in the accumulation of PrP^Sc^, and it ultimately results in the production of ROS, leading to decreased ATP building capacity of the mitochondria, and the autophagic signals are initiated. Increased autophagic flux has been shown to be useful for the degradation of misfolded prion proteins. The autophagic flux mediated by p62/LC3 plays an important role in the detachment of NRF2 from Keap1 and it ultimately translocates to the nucleus. After nuclear translocation, NRF2 triggers the induction of antioxidant genes and detoxifying enzymes. Finally, these antioxidant genes and detoxifying enzymes produced after NRF2 activation will decrease the oxidative stress to enhance survival.

NRF2 is a dynamic cellular transcription factor responsible for the regulation and subsequent expression of those antioxidative proteins which protect the cell against oxidative stress initiated by the production of ROS or neuroinflammatory events (Figure 3; Itoh et al., 1997). A significant increase in the NRF2 expression at the mRNA level in the two most common subtypes of sCJD, MM1 and VV2 cases, indicated a high cellular demand for the NRF2 antioxidant activity. The ubiquitin-proteasomes system is responsible for the continuous ubiquitination and degradation of NRF2 in the cytoplasm. Tahir et al. (2018) recently reported a significant decrease in the expression of degraded NRF2 in the two subtypes of sCJD, termed as MM1 and VV2, in comparison to the similar age control brains. The release of transcription factor NRF2 from Keap1 leads to its phosphorylation. Both the MM1 and VV2 subtypes of sCJD had significantly increased the expression of phosphorylated NRF2 in comparison to the age-matched control brains. Additionally, this increase was also observed at the protein level in both the subtypes of sCJD as compared to the age-matched control brains (Tahir et al., 2018).

The nuclear translocation of NRF2 and the eventual conversion to phosphorylated NRF2 support the transcription of an ARE. The ARE response element induces its target genes, such as heme oxygenase 1 (HMOX-1), NADPH quinine peroxidase, glutathione Stransferase (GST), glutathione cysteine Iigases (GCLs), peroxiredoxins (PRDX) and glutathione peroxidase, in the nucleus (Venugopal and Jaiswal, 1996; Itoh et al., 1997). Tahir et al. (2018) examined the expression of some selected members of the ARE response family including PRDX3, HMOX-1 and GSTM2 in both the subtypes of sCJD patients. HMOX-1 and PRDX3 showed an enhanced mRNA expression only in the VV2 subtype brains, whereas the GSTM2 showed a significantly enhanced expression in both MM1 and VV2 subtypes in comparison to their healthy age-matched control brains. At the protein level, the HMOX-1 protein showed a highly significant increase in the VV2 subtype brains, and the PRDX3 protein showed a significant elevation in both the subtypes of sCJD brains. The GSTM2 protein expression was significantly increased only in the VV2 subtype brains of sCJD, indicating that GSTM2 was an important marker of VV2 brain samples. These results showed that the ARE machinery is activated in the cerebellum of sCJD individuals to combat the stress produced by ROS (MM1 and VV2; Tahir et al., 2018). The above results indicate that the NRF2 activation occurs in prion diseases, but it might be insufficient to rescue patients from the disease (Tahir et al., 2018). Thus, p62-modulated NRF2 activation might be used as a possible therapy for these diseases to simultaneously target autophagy, ROS, and mitochondrial dysfunction.

## Activators of NRF2

There are several compounds which are able to react with and modify the activity of cysteine sensors of Keap1, subsequently initiating the macromolecular changes, which prevent or suppress the ubiquitination and degradation of the NRF2 and leads to its accumulation and nuclear translocation. Majority of the small molecular pharmacological activators of the NRF2 are electrophilic in nature and they are structurally dissimilar (Table 1). They contain cyano-enones such as TBE-31, which is a tricyclic compound possessing two highly reactive electron-withdrawing Michael acceptor groups (Liby et al., 2008). The TBE-31 cyano-enone binds to its targeted site in a strong reversible covalent manner, and it also represents one of the most efficient NRF2 activators recognized so far. Another NRF2 activator is sulforaphane (SFN), which is also known as sulphoraphane. The SFN is an isothiocyanate extracted from the cruciferous vegetables such as broccoli, Brussels sprouts and cabbage. It is one of the major naturally available NRF2 activators known (Zhang et al., 1992). The Fumaric acid esters, also known as dimethyl fumarate, can also stabilize the NRF2 activity (Spencer et al., 1990). These esters have been successfully used for the management of conditions such as psoriasis since a long time (Kolbach and Nieboer, 1992). The orally available marketed formulation of dimethyl fumarate, also known as the BG-12, has been recently accepted for the cure of a neurodegenerative disease, multiple sclerosis (Linker et al., 2011). Dimethylformamide (DMF) is an organic chemical compound with the formula of (CH3)2NC(O)H, and it is used for the treatment of relapsing multiple sclerosis (Fox et al., 2014). DMF activates NRF2 through the modification of the Keap1 sensor, which is attached to the NRF2 in the cytoplasm. DMF possesses low effectiveness and specificity for the activation of NRF2, and this prevents its broad use for neurodegenerative disorders. There are a number of other drug-like molecules with a similar mechanism of action as DMF, but they directly interfere with the Keap1/NRF2 interaction and show more prospects for targeting NRF2 in neurodegenerative diseases (Bertrand et al., 2015; Quinti et al., 2016).

**Table 1:** List of commonly used nuclear factor erythroid 2-related factor 2 (NRF2) activators.

Name	Nature	Intended usage	Reference
TBE-31	Tricyclic compound	Induction of phase-2 cytoprotective pathways	Liby et al. (2008)
Sulforaphane	Organosulfur compound	Major protective phytochemical against neurodegeneration	Zhang et al. (1992)
DMF	Methyl ester of fumaric acid compound	Stop the relapse of multiple sclerosis	Linker et al. (2011)
tBHQ	Quinone compounds	Aryl hydrocarbon receptor ligand	Probst et al. (2015)
Resveratrol	Natural phenol compound	Antioxidant and neuroprotective	Yadav et al. (2018)
tHIQ	Tetrahydroisoquinoline compound	Cytoprotective oxidative stress response	Richardson et al. (2015)

A number of other important molecules known to stabilize the activity of the NRF2 pathway are quinone compounds known as tert-butylhydroquinone (tBHQ; Li et al., 2005). The tBHQ compounds, such as triterpenoids bardoxolone methyl (Dinkova-Kostova et al., 2015) and RTA 408 (Probst et al., 2015), are currently in the clinical trial phase towards the development of universal treatment for NRF2 activation. The recent emergence of resveratrol-loaded solid lipid nanoparticles (R-SLNs) gave a new perspective to therapeutic neuroscience and it has been demonstrated that the R-SLNs significantly improved the oxidative stress-induced damage by modulating the Mn-SOD activity. Furthermore, it decreased hypoxia-inducible factor 1α (HIF-1α) levels and increased the NRF2-HO-1 activity to rescue vascular dementia rats from cognitive decline (Yadav et al., 2018). Besides these compounds, several non-electrophilic activators of NRF2 have been identified, such as tetrahydroisoquinoline (THIQ) and naphthalene (Richardson et al., 2015).

On one hand, the NRF2 activation can represent an outstanding pharmacological approach for the diseases where the ROS-mediated mitochondrial dysfunctions are primary features of the diseases, including prion diseases. Contrary to that, it is also very realistic to look at the possible toxicity that these molecules may produce and the dosage regimen that should be selected (de Zeeuw et al., 2013). Additionally, further molecular research is required to find out the harmful effects of NRF2 activation in neurodegenerative diseases. There are several reports that the NRF2 activation leads to further progression of tumor by increasing the number of tumor cells. This cytoprotective and pro-survival activity in the cells might be related to the reduced production of ROS species in the cells, which will ultimately promote the survival of tumor cells in certain situations. The gain of function in the NFE2L2 mutations or loss of function in the KEAP1 mutations are well established factors in different tumors. The reduced expression levels of Keap1 or the increased expression of NRF2 protein have been linked with the grave prognosis in several tumors (Solis et al., 2010). For the reasons mentioned above, one must be careful in designing NRF2-based therapeutic approaches (Sporn and Liby, 2012).

## Activation of P62-Keap1-NRF2-ARE Pathway and Selective Autophagy Induction as Possible Therapy for Prion Diseases

Several neurodegenerative diseases are outstanding candidates for NRF2-targeted therapies such as AD, Parkinson’s disease, ALS disease, frontal and temporal lobes affecting frontotemporal dementia disease, autosomal recessive inherited disorder—Friedreich’s ataxia, fatal genetic disorder, HD and progressive debilitating prion diseases. All of the above-mentioned disorders are predominantly characterized by the production of excessive amounts of ROS leading to oxidative stress and accumulation of unfolded or misfolded protein aggregates within the brain. Therefore, targeting ROS and misfolded proteins is always measured as a significant therapeutic aim for these diseases (Burchell et al., 2010). The greatest ability of the NRF2 signaling cascades in regulating the genes associated with the antioxidant defense mechanisms, autophagic mechanisms and ubiquitin-proteasome system activation leading to the degradation of misfolded proteins has been attracting the attention of many research groups. The use of NRF2 activators as a curative strategy for several neurodegenerative disorders has been reviewed by Hensly and Johnson (Hensley and Harris-White, 2015; Johnson and Johnson, 2015).

Autophagy is also known as a self-degradative process, vital for the balance of the source of energy at important moments in development and predominantly in response to nutritional stress. Besides this, autophagy also performs the housekeeping role in the removal of the misfolded aggregated proteins, and the clearance of damaged and impaired organelles, such as energy storing organelle mitochondria, Ca^2+^ storing organelle endoplasmic reticulum, and peroxisomes. Furthermore, it also eliminates the intracellular pathogens (Mizushima and Komatsu, 2011; Ichimura et al., 2013). Thus, the autophagic mechanism is generally measured as a pro-survival mechanism, although the impaired autophagy has been linked to non-apoptotic cell death. Reduced autophagic flux and defects in the endosomal/lysosomal functions may lead to the pathogenesis of prion diseases. It has been demonstrated that prion-infected brain tissues had enhanced levels of a multifunctional protein, Galectin-3, which participates in the intervention of inflammatory cellular reactions. Furthermore, the quantities of the lysosomal-associated membrane protein 2 (LAMP-2) were markedly reduced in the prion-infected galectin-32/2-mice. Low mRNA levels of Beclin-1, a mammalian ortholog of the yeast autophagy-related gene 6 (Atg6) and Atg5, showed impairment of the autophagic mechanism (Mok et al., 2007). Moon et al. (2016) further established that an improved or enhanced autophagy induced by a herbal formulation, ginsenoside-Rg3, significantly improved the human prion protein-mediated neurotoxic signaling events and alleviated the mitochondrial impairment seen in SK-N-SH cells infected with the prion peptide (PrP106–126).

Treating prion-infected mice with an autophagy-inducer rapamycin showed a prolonged survival period in comparison to the vehicle-treated control mice (Cortes et al., 2013). Similarly, autophagic flux protected the yeast cells against the *de novo* formation of PrP^Sc^ in a yeast model of prion diseases, and improved autophagy resulted in the degradation of the pathogenic scrapie proteins PrP^Sc^ (Speldewinde et al., 2015). It has been established that autophagy-inducer rapamycin treatment reduced the levels of aggregated PrP^Sc^ in an mTOR-dependent manner. The rapamycin therapy improved the mitochondrial dysfunctions, and neuroinflammatory and neurodegenerative events in mice model of the neurological disorder called Leigh syndrome (Johnson et al., 2013). These encouraging effects of the rapamycin therapy may be related to the indirect inhibition of protein synthesis machinery, which eventually preserves the ATP levels within the cell (Zheng et al., 2016). Furthermore, the emergence of small molecules for the induction of autophagy in mTOR-dependent and mTOR-independent manners has been considered vital for the treatment of several diseases (Sarkar et al., 2007; Chu et al., 2013). It suggests that there might be the possibility that mTOR-dependent and mTOR-independent small molecules could be used as a combinatory treatment in patients with prion diseases. More wide-ranging *in vivo* investigations, explaining the state of small molecules as a treatment policy for the patients affected with prion diseases, are important in this aspect.

A large number of recent molecular data suggest the significance of selective autophagy. Selective autophagy degrades the aggregated proteins termed as aggrephagy, and it also removes the unnecessary or impaired mitochondria termed as mitophagy. Selective autophagy can eliminate the invading bacteria known as xenophagy via the ubiquitin proteasome signaling pathways (Deretic and Levine, 2009; Kirkin et al., 2009; Youle and Narendra, 2011). The Keap1-NRF2 signaling cascades and autophagy are both implicated in the response against oxidative-stress, metabolic stress, and innate cellular immunity. Alternatively, the interplay between the important NRF2 influencing protein Keap1 and autophagy remains largely unknown. Recently, it has been demonstrated that the NRF2 modulates chaperone-mediated autophagy through the regulation of LAMP-2a in different human and mouse cell lines (Pajares et al., 2018). Similarly, Tang et al. (2018) demonstrated that the NRF2 mediates the expression of cochaperone Bcl-2-associated athanogene 3 (BAG3) and autophagy adaptor proteins NBR1, NDP52 and sequestosome 1/p62 and tau clearance in wild type and NRF2 knockout mice in an age-dependent manner. Ichimura et al. (2013) showed that the phosphorylation of autophagy-adaptor protein p62 clearly increased the p62’s binding potential for the Keap1, an adaptor protein belonging to the Cul3-ubiquitin E3 ligase complex which is vital for degrading the NRF2 protein. In addition, the p62 phosphorylation triggered the expression of cytoprotective targets of NRF2 such as the ARE. The p62 is accumulated on selective autophagic cargos, for instance ubiquitinated organelles, and then it is phosphorylated in an mTORC1-dependent manner, thereby showing the coupling of the Keap1 and NRF2 systems with autophagy (Ichimura et al., 2013). Recently, Khan et al. (2017) showed that the treatment of N2a cells with prion peptide 106–126 triggered autophagy, which was evident by a decrease in the p62 levels and increase in the LC3-II levels up to 24 h, and then autophagy was decreased in a time-dependent manner from 24 h up to 48 h. This shows the impairment in autophagy during prion infection. Homma et al. (2014) also showed high levels of p62 phosphomimic form and LC3-II in the brains of 22L-infected mice and 263K-infected hamsters. Furthermore, phosphomimic p62 enhanced the activity of ubiquitin-binding system to degrade PrP^Sc^ (Homma et al., 2014). We propose that simultaneous selective targeting of p62 and NRF2 might prove beneficial to reduce prion-induced autophagic defects and oxidative stress on one hand while it can decrease the levels of PrP^Sc^ on the other hand, which is sure to further decrease the oxidative stress to enhance survival.

## Concluding Remarks and Future Directions

Prion diseases, also called as TSEs, are a unique group of terminally debilitating neurodegenerative disorders characterized by a long-term progressive deterioration leading to motor paralysis and complete disability in affected humans. To date, there is no efficient therapy available for prion diseases regardless of the extensive research efforts dedicated to this disease over the past few years. Symptomatic remedies are available for some neurodegenerative diseases, such as Parkinson’s diseases and HD, but the therapeutic benefits are temporary and limited. Despite the fact that the causative factors and clinical manifestations are different for each neurodegenerative disease, their cellular pathogeneses share common underlying factors, such as the production of excessive levels of ROS, largely due to mitochondrial dysfunction, neuroinflammation, and disturbances in protein homeostasis. This opens an entirely new and exciting possibility for developing a general treatment, targeting these common drivers for the rescue of neurodegeneration.

NRF2 is a transcription factor controlling a major endogenous cellular defense mechanism against the oxidative and nitrositive stress and inflammation, and it plays a vital role in the maintenance of mitochondrial homeostasis and cellular proteostasis, suggesting it to be a potentially valuable therapeutic target against neurodegeneration. During stress conditions, NRF2 activates the transcriptional up-regulation of a large network of cytoprotective genes, allowing adaptation and survival. A recent study by Tahir et al. (2018) showed that the levels of the important ARE defense mechanism proteins HMOX-1, PRDX3, and GSTM2 were higher in the brain of sCJD patients in comparison with non-diseased controls, suggestive of the partial activation of NRF2-mediated defense signaling. However, this activation is insufficient to block the progressive neurodegeneration. Although the oxidative stress and neuroinflammatory events are the pathological hallmarks of prion diseases, a therapeutic role of NRF2 signaling has not been tested yet, perhaps due to the abrupt progressive nature and complexity of disease pathogenesis. Nevertheless, there are several recent publications which demonstrate the efficacy of NRF2 activators in other neurodegenerative diseases such as ADs, Parkinson’s disease and ALS disease (Jazwa et al., 2011; Kanno et al., 2012; Ahuja et al., 2016; Liu et al., 2016).

Autophagic defects and impaired autophagic flux are common features of prion diseases. P62-mediated activation of the NRF2 has recently gained the attention of researchers working on neurodegenerative diseases. Active autophagy can be protective in reducing the level of oxidative stress in a two-pronged manner—one would be via decreasing the amount of misfolded prions, and second would be the detachment of NRF2 from Keap1 (triggered by phosphorylated p62), which will ultimately translocate the NRF2 to the nucleus and activate the antioxidant defense mechanisms.

In summary, the p62-Keap1-NRF2-ARE signaling pathway may provide an exciting and new therapeutic alternative to improve the disease pathology in common neurodegenerative diseases. Since the pharmacological NRF2 activation targets the broad mechanisms of disease, all the neurodegenerative conditions would be eligible for therapy.

## Author Contributions

SS wrote the manuscript. DZ and LY conceived the idea for the study. NS and MM helped with figure preparation and TH critically reviewed the manuscript before final submission.

## Conflict of Interest Statement

The authors declare that the research was conducted in the absence of any commercial or financial relationships that could be construed as a potential conflict of interest.
